# Comparison of Metal Nanoparticles (Au, Ag, Eu, Cd) Used for Immunoanalysis Using LA-ICP-MS Detection

**DOI:** 10.3390/molecules26030630

**Published:** 2021-01-26

**Authors:** Marcela Vlcnovska, Aneta Stossova, Michaela Kuchynka, Veronika Dillingerova, Hana Polanska, Michal Masarik, Roman Hrstka, Vojtech Adam, Viktor Kanicky, Tomas Vaculovic, Marketa Vaculovicova

**Affiliations:** 1 Department of Chemistry and Biochemistry, Mendel University in Brno, Zemedelska 1, CZ-613 00 Brno, Czech Republic; marcelavlcnovska@seznam.cz (M.V.); masarik@med.muni.cz (M.M.); vojtech.adam@mendelu.cz (V.A.); 2 Department of Pathological Physiology, Faculty of Medicine, Masaryk University/Kamenice 5, CZ-625 00 Brno, Czech Republic; hana.polanska@gmail.com; 3 Central European Institute of Technology, Brno University of Technology, Purkynova 123, CZ-612 00 Brno, Czech Republic; 4 Department of Chemistry, Faculty of Science, Masaryk University, Kamenice 753/5, CZ-625 00 Brno, Czech Republic; anetastossova@gmail.com (A.S.); 358018@mail.muni.cz (M.K.); nikadilli@gmail.com (V.D.); viktork@chemi.muni.cz (V.K.); vaca_777@yahoo.com (T.V.); 5 Research Centre for Applied Molecular Oncology, Masaryk Memorial Cancer Institute, Zluty kopec 7, CZ-656 53 Brno, Czech Republic; hrstka@mou.cz

**Keywords:** protein p53, dot-blot, antibody

## Abstract

Immunochemical methods are used not only in clinical practice for the diagnosis of a wide range of diseases but also in basic and advanced research. Based on the unique reaction between the antibody and its respective antigens, it serves to specifically recognize target molecules in biological complex samples. Current methods of labelling antibodies with elemental labels followed by detection by inductively coupled plasma mass spectrometry (ICP-MS) allow detection of multiple antigens in parallel in a single analysis. Using the laser ablation (LA) modality (LA-ICP-MS), it is also possible to monitor the spatial distribution of biogenic elements. Moreover, the employment of metal nanoparticle-labeled antibodies expands the applicability also to molecular imaging by LA-ICP-MS. In this work, conjugates of model monoclonal antibody (DO-1, recognizing p53 protein) with various metal nanoparticles-based labels were created and utilized in dot-blot analysis in order to compare their benefits and disadvantages. Based on experiments with the p53 protein standard, commercial kits of gold nanoparticles proved to be the most suitable for the preparation of conjugates. The LA-ICP-MS demonstrated very good repeatability, wide linear dynamic range (0.1–14 ng), and limit of detection was calculated as a 1.3 pg of p53 protein.

## 1. Introduction

Up to recently, inductively-coupled laser ablation plasma mass spectrometry (LA-ICP-MS) has definitely not been promoted in bio-applications, simply because of its primary orientation on detection of sample elemental composition, whereas (bio)analytical fields are interested more in molecular composition and interactions. Inductively coupled plasma mass spectrometry (ICP-MS) is an extremely sensitive method for the determination of elements in samples. Its main advantages are the speed of analysis and the ability to determine multiple elements within a single analysis. In combination with laser ablation (LA-ICP-MS) it allows not only the determination of elements, but also their spatial distribution in the sample, and its sensitivity makes it possible to detect trace elements in almost any matrix.

However, a dramatic change has taken place when imaging mass cytometry (CyTOF) was developed and introduced [1]. In CyTOF analysis, the antigens are immunologically stained in a familiar manner, except that the probes are tagged with metal isotopes instead of fluorophores [2]. The biggest advantage of imaging mass cytometry is that a single tissue sample can be imaged for 40+ markers simultaneously. This multiplexing capability given by ICP-MS is significantly improved compared to optical fluorophores which are extremely useful particularly in disease (e.g., cancer) and biomarker research (isotope-labeled antibodies against a number of biomarkers are simultaneously used to explore their expression within the diseased cells/tissues).

Historically, antibodies are the most commonly used group of biorecognition elements. Therefore, antibodies labelled by an elemental tag—chelate binding rare earth elements [3,4] enabled to detect specific proteins (e.g., p53 [5], ferroportin [6], MMP-11 [7], etc.) in levels of pg [8] by LA-ICP-MS. Also, currently available commercial CyTOF instruments rely on the use of antibodies [9,10]. So far, most LA-ICP-MS/CyTOF studies apply single-atom chelates (e.g., MeCAT or Maxpar). However recently, it was demonstrated that utilization of nanoparticles leads to improvement in limits of detection by an order of magnitude compared to MeCAT due to the high number of metal atoms within a single nanoparticle [8].

This method has great potential in imaging biological samples such as cells and tissues. It brings information not only about the quality and quantity of detected analytes but also about their distribution. Along with proteins, it is also possible to monitor the natural distribution of biogenic elements. This may be interesting to study the tumor microenvironment or analysis of metal-containing proteins. Or the pathological occurrence of elements such as accumulated heavy metals in tissues.

In this work, antibody conjugates with several metal nanoparticles (Au (AuNPs), Ag (AgNPs), Eu (EuNPs), and CdTe quantum dots (CdTe QDs)) were tested to evaluate their benefits and disadvantages in the immunochemical analysis (dot-blot assay) coupled to LA-ICP-MS using p53 protein as a model analyte. This arrangement is beneficial as only 0.5 μL of antigen can be applied on the membrane. 0.5 μL is 100x less than, for example, in comparison with standard ELISA method, which requires approximately 50 μL of sample to 96-well plate with comparable LODs (Table 1)

## 2. Results and Discussion

The p53 protein serves as a model antigen mainly due to the possibility of using a very specific antibody DO-1. This antibody targets the N-terminus of the human p53 protein between amino acids 20 and 25, at a site where the amino acid sequences of human and murine p53 differ by only amino acid 21. Aspartic acid in humans is replaced by glycine in mice. Despite the significant similarity of the binding site, the DO-1 antibody is highly specific and recognizes only the human p53 protein without cross-reactivity between species [17,18].

Conjugates of AuNPs, AgNPs, EuNPs, and CdTe QDs with DO-1 antibody were prepared. The mechanism of binding via the primary amine of lysine residues was chosen for conjugation of antibodies with nanoparticles.

Abcam company (Cambridge, UK), which supplies gold and europium nanoparticles, does not further specify the binding mechanism. Particles from Cytodiagnostics company (Burlington, ON, Canada) were delivered as NHS activated. A 5 kDa long polyethylene glycol linker was attached to the surface of the silver nanoparticles via a thiol, at the end of which NHS-ester was linked allowing rapid binding of the protein to the primary amine. Antibodies were also bound to carboxylated CdTe QDs supplied by Themofisher Scientific (Waltham, MA, USA) by a similar mechanism. The reaction of the carboxyl with EDC and NHS reagents resulted in the formation of a stable NHS-ester intermediate, which was then able to react with the primary amine to form a solid amide bond [19].

### 2.1. Laser Beam Fluence Optimization

**Figure 1:**
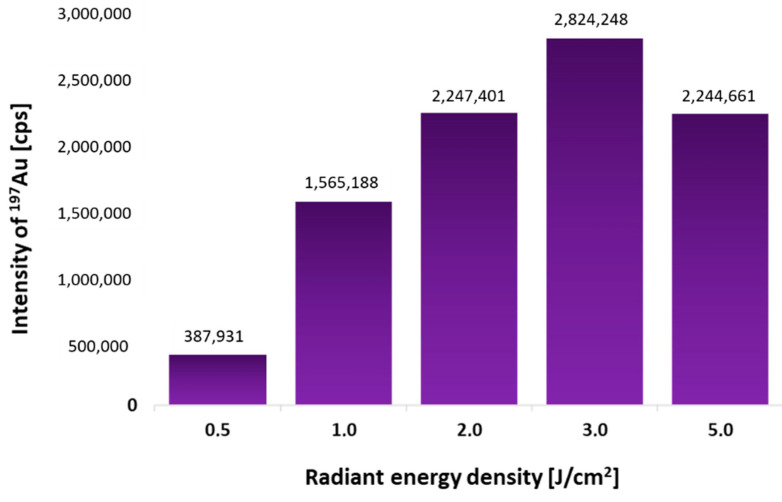
Dependence of the measured Au intensities on the various radiant energy densities, while the amount of antigen applied to the PVDF membrane was identical.

Based on our previous experiments and experience [8], the laser beam size of 150 μm and the sample feed rate of 500 μm/s were chosen for the fastest possible analysis. Only the laser beam fluence was optimized. Five similar 0.5 μL spots of p53 protein (4 ng/μL) were spotted onto the PVDF membrane. After dot-blot using AuNPs-DO-1 conjugate, each spot was ablated under different laser beam fluence (0.5–5 J/cm^2^). The dependence of the measured Au intensities on the used laser beam fluence is shown in Figure 1. It was found that the most suitable is to use a laser beam fluence of 3 J/cm^2^, as the signal is highest under these conditions.

### 2.2. Repeatability of the Analysis

**Figure 2:**
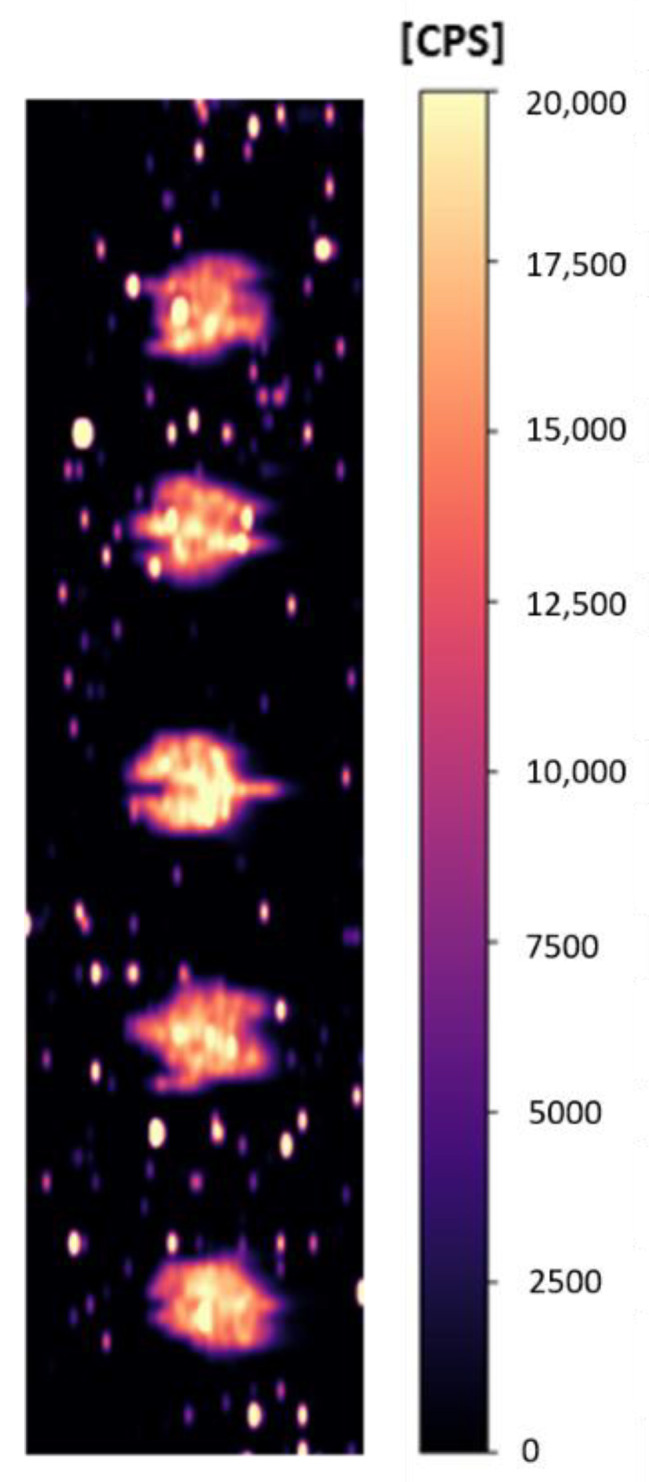
Repeatability of measurement (five 0.5 µL spots of p53 (4 ng/ μL) using AuNPs-DO-1).

Due to the very small sample volume that was pipetted onto the membrane (0.5 μL), the repeatability of the method had to be verified. Five similar 0.5 μL spots of protein p53 (4 ng/μL) were spotted on the PVDF membrane and measured. The obtained data is shown in Figure 2.

Measured data are shown in Table 2. The relative standard deviation was 3%, which is satisfactory. The analysis time for this membrane was 60 min. From this, we can evaluate that during the whole measurement there were no signal fluctuations in time, due to the instability of the ICP-MS or the energy of the laser radiation, and the Au intensities are constant throughout the measurement.

### 2.3. Verification of Antibody Activity after Binding to Various Nanoparticles

After the conjugation, it was necessary to verify that the conjugate was formed successfully and that the antibody activity was remained. The binding of the antibodies to the nanoparticles via the primary amine is random, and therefore there is a possibility of antibody binding through the active site if it contains lysine. Nevertheless, the tertiary structure may be deformed if it binds near the active site [20].

Selected nanoparticles were conjugated with p53 protein-specific antibody (DO-1). Verification of the antibody-nanoparticle conjugate functionality was performed by dot-blot analysis. A concentration line of antigen standard was applied to the PVDF membrane. 0.5 μL of p53 protein sample with a concentration in the range of 0.20–20.00 μg/mL. It means that the absolute amount of protein per spot applied at membrane was in the range of 0.10–10.00 ng.

In all experiments, performed using commercial kits, 0.13 μL of the conjugate was diluted with antibody buffer to a total volume of 1 mL. For quantum dots, 40 μL of conjugate diluted to a total volume of 1 mL was needed to be used for achieving comparable results.

**Figure 3:**
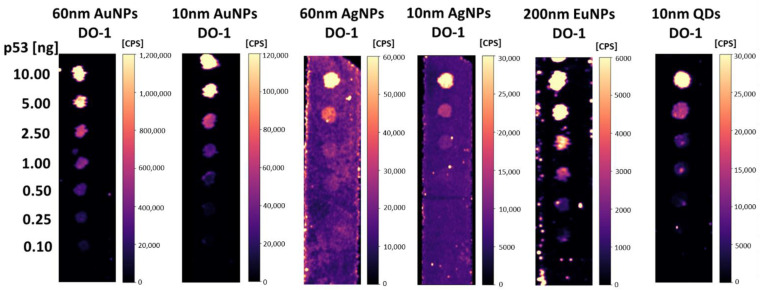
LA-ICP-MS dot-blot analysis of p53 protein standard (total protein amount 0.10–10.00 ng) using DO-1 antibody conjugates with Au, Ag, Eu, and QD nanoparticles.

Antibody conjugation was successful for all nanoparticles. The antibody did not lose its ability to bind antigen, as shown in Figure 3. It has to be noted, that it is not possible to compare the signal intensities of individual isotopes mainly due to the fact that each isotope has a different abundance (^197^Au—100%, ^153^Eu—52%, ^107^Ag—52%, ^111^Cd—13%), and different ionization energy (9.2 eV for Au, 9.0 eV for Cd, 7.6 eV Ag, 5.7 eV Eu). Hence, different elements have a different response in ICP-MS. Therefore, it is not possible to compare the signal intensities of different elements. The least suitable antibody-nanoparticle conjugates were those with 10 nm and 60 nm AgNPs. These conjugates showed the highest background signal all over the blotting membrane. The other conjugates appeared to be suitable for immunodetection. However, there would be probably some amount of nanoparticles not completely covered by the antibodies. It was, therefore, necessary to verify the contribution of non-specific sorption of bare (or incompletely covered) nanoparticles.

### 2.4. Contribution of Bare (Incompletely Covered) Nanoparticles to Non-Specific Sorption

**Figure 4:**
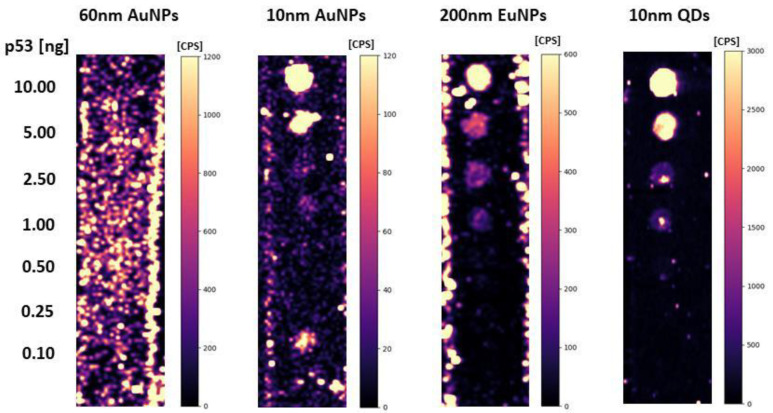
LA-ICP_MS dot-blot analysis of p53 protein standard (total protein amount 0.10–10.00 ng) using Au, Ag, Eu, and QD nanoparticles without antibody presence.

QDs and EuNPs without the presence of the antibody were found to have high non-specific sorption, which is only one order of magnitude lower than the specific sorption (ensured by DO-1 antibody) as concluded from intensity values in Figure 3 and Figure 4. In the case of 10 nm and 60 nm AuNPs, the non-specific sorption was three orders of magnitude lower than the specific sorption. For 60 nm AuNPs, non-specific sorption was observed on the entire membrane surface, while for 10 nm AuNPs non-specific sorption was observed for individual spot samples. Such non-specific sorption can then be easily subtracted from the positive control (Figure 4).

In blotting techniques, we need to determine the trace amount of antigen and the spatial resolution is not the most critical parameter. Therefore, it is appropriate to use larger nanoparticles (60 nm AuNPs), which have higher response and thus, lower limits of detection can be achieved. Smaller nanoparticles (10 nm AuNPs) are likely to be more suitable for the immunohistochemical and immunocytochemical determination of antigens. Large nanoparticles may have problems with penetrating the cytoplasmic membrane of cells. Their steric effect can then lead to lower quality of spatial resolution. Therefore, 10 nm AuNPs were selected for the following experiments. It has also been experimentally verified that the conjugates are stable for more than one year when stored correctly, i.e., at 4 °C.

### 2.5. Linearity of Calibration Dependence

**Figure 5:**
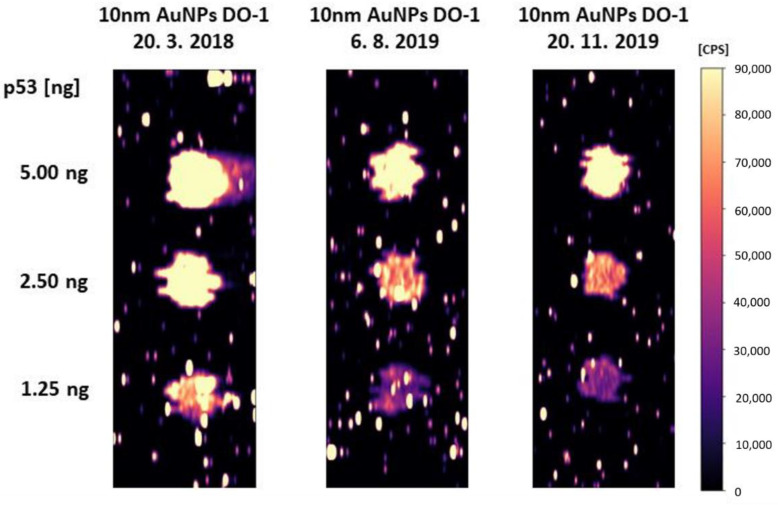
Conjugates stability: dot-blot performed with differently old conjugates (dates of conjugation are marked in the picture).

After optimizing the conditions and verifying the repeatability, two calibration series of different ranges of antigen amount were prepared (0–1.2 ng and 0–14 ng of p53). The measured Au intensities corresponded to different amounts of analyte applied to the membrane, even though the same amount of nanoparticle-antibody conjugate was applied to the membrane. In Figure 5, the difference in the slopes of the two calibration curves can be observed. The slope of Calibration curve 1 (blue trace) with a lower amount of the p53 is about 1.5 times higher than the slope of Calibration curve 2 (purple trace). The intercepts were statistically tested against zero, and they were found to be equal to zero. The detection limit of 1.3 pg was calculated using the slope of Calibration curve 1.

### 2.6. Dot-Blot Analysis of p53 Protein in a Protein Mixture

After evaluation of the method using a simple one-protein mixture, the complexity of the sample was increased. For a model example of a more complex matrix, a mixture of known protein composition was used for analysis. The ladder, a mixture of proteins of different sizes used as a size marker and weight standard in SDS-PAGE, was enriched by the addition of a p53 protein. The size of the 12 highly purified proteins in the mixture ranged from 10–250 kDa, and their total concentration reported by the supplier is 2 mg/mL. This sample was mixed 1:1 with p53 protein at a concentration of 20 μg/mL and subsequently diluted to reach the required concentration.

**Figure 6:**
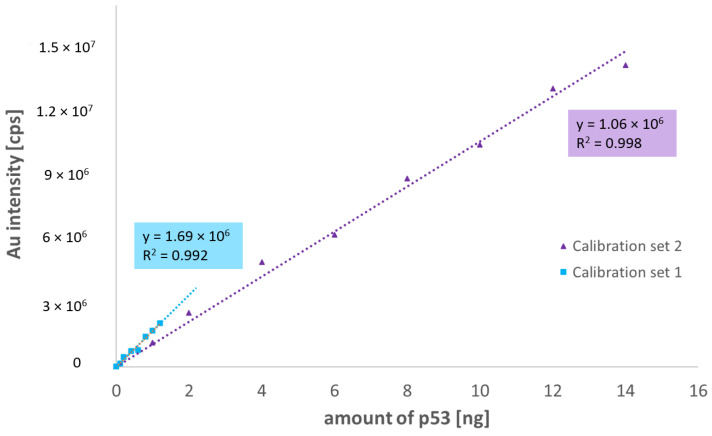
p53 calibration curves 0.1–1.4 ng—blue, 1–14 ng—purple.

As expected, the non-specific sorption slightly increased and therefore, we tried to eliminate it by blocking the potentially exposed metal surface, which is highly probably causing the non-specific sorption. We used electrostatic adsorption of 1% BSA, which is a commonly used strategy in blotting techniques for blocking the membrane. By additional blocking of not only the membrane but also of the particle uncovered surface, double blocking effect was reached. Four identically prepared membranes (Figure 6) reacted with AuNPs-DO-1, BSA-blocked nanoparticles-antibody conjugates (AuNPs-DO-1-BSA), bare AuNPs, and bare AuNPs blocked by BSA (AuNPs-BSA).

It was found that the non-specific sorption of AuNPs was 5.4%. AuNPs-BSA were found to have higher non-specific sorption compared to bare AuNPs, which may be caused by electrostatic interaction of BSA with proteins within the sample. The non-specific sorption of AuNPs-BSA increased to almost 50% in the case of the most concentrated sample.

Interestingly, AuNPs-DO-1-BSA exhibited lower measured intensities and in comparison with AuNPs-DO-1.

This may have several explanations. Either the BSA covers the surface of the nanoparticle where the antibody did not bound, thus eliminating non-specific interaction with the sample, or it electrostatically interacts not only with the nanoparticle but also with the antibody. Thus, it is possible that it affects the active site of the antibody, and thus reduces the specific interaction, which is undesirable.

**Figure 7:**
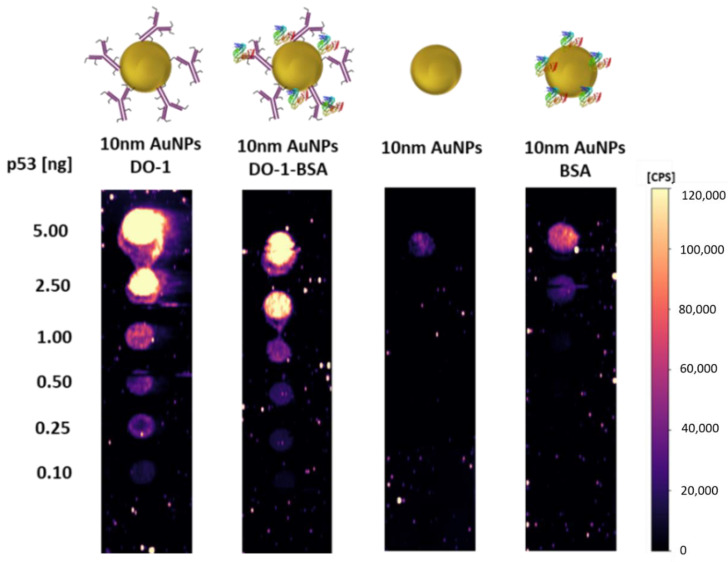
Dot-blot analyses of p53 using AuNPs-DO-1, AuNPs-DO-1-BSA, AuNPs, and AuNPs-BSA.

**Figure 8:**
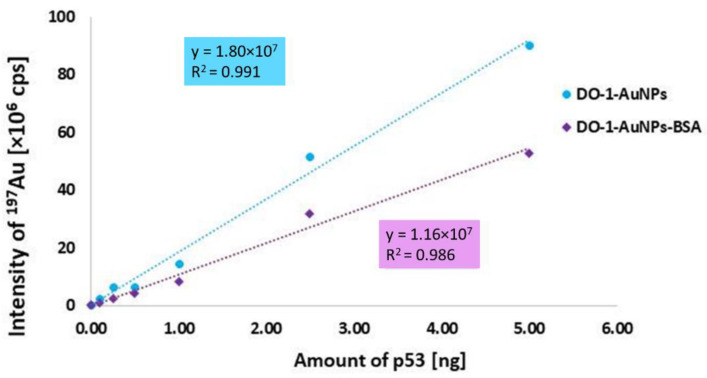
Calibration curves of p53 protein quantification using AuNPs-DO-1 and AuNPs-BSA.

Moreover, as shown in Figure 7, even though the use of AuNPs-DO-1-BSA provided lower specific as well as non-specific sorption, the sensitivity (expressed as calibration curve slope, Figure 8.) is increased in case of AuNPs-DO-1.

## 3. Materials and Methods

### 3.1. Chemicals

Unless stated otherwise, the chemicals were purchased from Sigma-Aldrich. All solutions were prepared using ultrapure Milli-Q water (purification system, Millipore, Bedford, MA). For conjugation was used anti-p53 antibody DO-1 obtained from Masaryk Memorial Cancer Institute in Brno. Recombinant Human p53 protein ab43615 Abcam, UK served as a model sample and Unstained Protein Ladder, Broad Range (10–250 kDa) New England BioLabs, USA was used for simulation of a complex sample.

### 3.2. Nanoparticles

60 nm AuNPs → GOLD Conjugation Kit (60 nm, 20OD) ab188216 → Abcam, UK

10 nm AuNPs → GOLD Conjugation Kit (10 nm, 20OD) ab201808 → Abcam, UK

200 nm EuNPs → Europium Conjugation Kit ab269889 Abcam, UK

10 nm AgNPs → NHS-Activated Silver Nanoparticle Conjugation Kit SKU: SN5K-10-1 Cytodiagnostics, Canada

10 nm AgNPs → NHS-Activated Silver Nanoparticle Conjugation Kit SKU: SN5K-60-2 Cytodiagnostics, Canada

QDs Qdot™ 655 ITK™ Carboxyl Quantum Dots Thermo Fisher Scientific, USA

### 3.3. The Composition of the Solutions

2× Blotting buffer: 25 mM Trizma base, 150 mM glycine, 10% (*v*/*v*) methanol

1× Blotting buffer: (50% (*v*/*v*) 2× blotting buffer with 40% (*v*/*v*) H_2_O and 10% (*v*/*v*) MetOH

Phosphate buffer saline (PBS): 2.7 mM KCl, 1.8 mM KH_2_PO_4_, 137 mM NaCl and 10 mM Na_2_HPO_4_, pH 7.4

Blocking buffer: 3% bovine serum albumin (BSA) in PBS

Antibody buffer: 0.1% BSA in PBS

PBS-T: 0.05% or 0.1% (*v*/*v*) Tween-20 in PBS

### 3.4. Preparation of Antibody-Nanoparticle Conjugates with AuNPs, AgNPs and EuNPs

Commercially available kits were used for the preparation of conjugates with gold, silver and europium nanoparticles. All necessary reagents, such as antibody diluent, reaction buffer and quencher, were supplied as part of the package. Antibody binding was performed according to the manufacturer’s protocol. For 10 nm AgNPs, the antibody was diluted with Antibody diluent to the concentration of 0.5 mg/mL compared to the recommended 3 mg/mL because such a concentrated antibody was not available. The manufacturer’s instructions were followed.

### 3.5. Preparation of Antibody-Nanoparticle Conjugates with QDs

150 μL of 0.1 μM carboxylated QDs were mixed with 5 μL of 1.2 mM NHS and 5 μL of 0.6 mM EDC. The mixture was incubated on a Thermomixer 5355 with gentle shaking and room temperature for 20 min. Then, 40 μL of 1.2 mg/mL DO-1 antibody was added and allowed to interact again with gentle shaking for 60 min.

### 3.6. Dot-Blot

The recombinant human p53 protein ab43615 (Abcam, Cambridge, UK) in the volume of 0.5 μL was applied as a sample at the blotting membrane, and dot-blot analyses were performed according to the procedure described previously [8]. The protein ladder (a purified mixture of proteins of various sizes corresponding to different molecular masses) spiked with p53 was used to mimic the complex sample.

### 3.7. LA-ICP-MS Analysis

LA-ICP-MS analysis of the dot-blot membranes was carried out by a laser ablation system LSX213 (CETAC, USA) emitting laser radiation of 213 nm with a pulse width of 4.2 ns and a quadrupole ICP-MS spectrometer Agilent 7900 (Agilent Technologies, Japan). The ICP-MS parameters were optimized with respect to the best S/N ratio, RSD and oxide ratio (ThO+/Th+) lower than 0.5% using glass reference material NIST610. The imaging of the dot-blot samples was performed using the following ablation parameters: a laser beam diameter of 150 μm, laser beam fluence of 3 J/cm^2^, a repetition rate of 10 Hz, a scan speed rate of 500 μm/s and a distance between individual lines of 150 μm.

The whole spot was ablated line by line, and the following isotopes were monitored: ^197^Au, ^153^ Eu, ^111^Cd, and ^107^Ag with an integration time of 0.1 s for each isotope.

### 3.8. Data Processing

The whole spot was ablated line by line, and the Au signal was measured. Then our lab-made software ILAPS was used for processing raw data (from the time-resolved signal to the matrix for 2D plots creation). The limit of quantification for signal intensity was calculated according to 10- fold of standard deviation of the gas level (without ablation). All intensities below the limit of quantification were set to zero [8]. Then the sum of intensities across the whole spot is was then calculated all 5 spots. Then the standard deviation was calculated from these five sums according to:

$$s=\sqrt{\frac{1}{N-1}\sum^{\;}{\left(x_{i}-\bar{x}\right)}^{2}}$$

## 4. Conclusions

It has been experimentally demonstrated that the utilization of the nanoparticle-antibody conjugates is suitable and beneficial for immunoassays due to low limits of detection, multiplexing ability, wide dynamic range, flexibility, and variability. From tested nanoparticles, 10 nm AuNPs were selected as those with optimal results. AgNPs exhibited very high background signal all over the blotting membrane and EuNPs and CdTe QDs displayed relatively high non-specific sorption. Finally, even though 60 nm AuNPs would be desirable for in vitro blotting analyses (dot-blot or western blot), experiments intending to monitor the spatial distribution of antigens in e.g., tissue sections would probably highly benefit from the performance of 10 nm AuNPs mainly due to the sterical hindrance of bigger nanoparticles. However, this hypothesis has to be verified. Other important aspect is that the use of nanoparticles reduces the consumption of antibodies, and if coupled with LA-ICP-MS, it is possible to use even a minimal amount of sample. The disadvantage of using nanoparticles is the increased non-specific sorption in more complex samples. Therefore, it is always necessary to select an appropriate negative control, verify non-specific sorption and minimize it.

## Figures and Tables

**Table 1:** Comparison of sample volume and p53 detection limits in different methods.

Method	Sample Volume	LOD *	Note	Ref.
ELISA Kit	50 μL	**8 µg/mL** 183 nM	Commercial Human p53 ELISA Kit (ab156027), Abcam UK records OD at 450 nm.	[11]
Bio-FET	20 μL	4.37 ng/mL **100 pM** *	Label-free field-effect transistor-based immunosensor.	[12]
LA-ICP-MS	0.5 μL	2.6 ng/mL 59.5 pM	Direct immunoassay using nanoparticle labelled antibodies.	This study
ELISA	50 μL	**0.44 ng/mL** 10 pM	PNPP was used as a substrate and absorbance was read at 405 nm.	[13]
ELISA	50 μL	**0.1 ng/mL** 2.3 pM	TMB was used as a substrate and absorbance was read at 250 nm.	[14]
Aptasensor	100 μL	**10 pg/mL** 229 fM	The catalytic activity of aggregated AuNPs increased chemiluminescence intensity.	[15]
p53 ELISA Kit	50 μL	**65 pg/mL** 1.49 pM	Commercial Human p53 ELISA Kit (ab171571), Abcam UK records the OD at 450 nm.	[16]

* bold units taken from the original work unified for comparison purposes.

**Table 2:** Repeatability of measurement: Measured Au intensities and calculated standard deviation and relative standard deviation.

p53 [ng]	Sum Au (AuNPs-DO-1) [CPS]
2	2,460,000
2	2,410,000
2	2,570,000
2	2,520,000
2	2,390,000
Average	2,470,000
SD	75,400
RSD [%]	3.05

## Data Availability

The data presented in this study are available on request from the corresponding author.
